# Improving childhood cancer medicines access in developing countries: Towards an implementation framework to inform the Global Platform for Access to Childhood Cancer Medicines for Nigeria

**DOI:** 10.1371/journal.pgph.0003275

**Published:** 2024-09-06

**Authors:** Otuto Amarauche Chukwu, Isaac Folorunso Adewole, Avram Denburg, Beverley M. Essue

**Affiliations:** 1 Institute of Health Policy, Management and Evaluation, Dalla Lana School of Public Health, University of Toronto, Toronto, Ontario, Canada; 2 Gynecological Oncology Unit, Department of Obstetrics and Gynecology, College of Medicine, University of Ibadan, Ibadan, Nigeria; 3 University College Hospital, Ibadan, Nigeria; 4 SickKids Research Institute, The Hospital for Sick Children, Toronto, Ontario, Canada; 5 Faculty of Medicine, University of Toronto, Toronto, Ontario, Canada; PLOS: Public Library of Science, UNITED STATES OF AMERICA

## Abstract

Children and adolescents in developing countries continue to be disproportionately affected by cancer and have significantly lower survival rates (30%) than their counterparts in high-income countries (80%). This disparity is driven by poor access to childhood cancer medicines. The World Health Organization and St. Jude Children’s Research Hospital launched the Global Platform for Access to Childhood Cancer Medicines to provide continuous supply of quality childhood cancer medicines to developing countries. As much movement has not been seen with the platform, this research aimed to develop a stakeholder-informed guidance to support effective implementation of the platform and maximize opportunities to deliver on its intended goals. This study was guided by the Consolidated Framework for Implementation Research (CFIR). Participants were recruited based on the stakeholder categories framework and included policymakers from the Ministry of Health, organizations implementing access to medicines programs in Nigeria, medicines logistics providers, and health professionals and personnel at service delivery points such as oncologists and pharmacists. Data collection involved key informant interviews using a pilot-tested semi-structured interview guide. Data analysis was done by thematic content analysis. Ethical approval was obtained from the National Health Research Ethics Committee of Nigeria and the Ethics Review Board of University of Toronto. The findings reveal critical insights spanning five domains of the CFIR framework, each contributing uniquely to understanding the multifaceted issues of childhood cancer medicine access with a view to understanding pathways to implementation of the platform. Successfully implementing the platform could entail a partner-driven approach, integration with existing programs to avoid fragmentation, supporting capacity strengthening at the primary care level, and engaging patients and communities. This information was used to suggest a nuanced implementation framework for the platform in Nigeria and similar settings which could be beneficial for improving access for children who desperately need childhood cancer medicines to survive.

## 1 Background

Cancer is a leading cause of death among children and adolescents aged 0–19 [1]. An estimated 400,000 new cancer cases are reported annually in this age group, with 90% occurring in low- and middle-income countries (LMICs) [1]. Children and adolescents in LMICs like Nigeria continue to be disproportionately affected by cancer and have significantly lower survival rates (30%) than their counterparts in high-income countries (80%) [2]. This has been attributed to, amongst other things, poor access to necessary treatments and the unaffordability of care that often results in delayed initiation of treatment as well as the abandonment of treatment and avoidable relapse [3]. Improving access to cancer medicines is a highly cost-effective and feasible strategy. It can improve survival [3], which is paramount in LMICs like Nigeria, where there continues to be a disproportionate burden of childhood cancer mortality [2].

The World Health Organization (WHO), in partnership with the St. Jude Children’s Research Hospital in the United States, launched the Global Platform for Access to Childhood Cancer Medicines in 2022 [4]. This six-year, USD 200 million investment is designed to provide a continuous supply of quality childhood cancer medicines to LMICs [4].

In countries with developing health systems, like Nigeria, medicines supply programs can fail due to inadequate considerations of peculiarities in healthcare service delivery, supply chain terrain, and ongoing inequities in access to medicines among those who stand to benefit most [5–7]. Such issues have been flagged as barriers to the effective implementation of existing programs such as, the Drug Revolving Fund (DRF) and the National Drug Policy, that failed to meet their desired objectives [5, 6]. Key inequities in access to medicines in Nigeria include long distance to treatment centers and catastrophic out-of-pocket expenses. Supply chain channels for medicines distribution remain chaotic and uncoordinated, leading to the pilferage and leakage of free medicines into private hands that put them up for sale in illegal markets [8, 9]. These issues increase the stock-out of these essential medicines, thus disenfranchising end users from access and contributing to health inequities [1]. Furthermore, most Nigerians (70.5%) pay out-of-pocket for healthcare, which increases the likelihood that households will accrue debt and forgo treatment [10, 11] from using required healthcare services. Approximately 4 in 10 people live in extreme poverty in Nigeria, and health care spending exacerbates this risk [12]. These issues are further compounded by the high level of circulating falsified, substandard, and falsely labelled drugs [13].

The Global Platform for Access to Childhood Cancer Medicines offers strong potential to overcome the existing inequities in access to affordable and lifesaving medicines for children with cancer in Nigeria. To deliver on the promise of this investment, there is a need for context-specific implementation frameworks to support the achievement of the desired objectives.

Implementation science focuses on promoting the systematic uptake of research findings and evidence-based practices into routine practice, thus improving the quality and effectiveness of health services and care [14]. It serves as a critical tool for bridging the gap between evidence and practice and is particularly useful for developing health systems with resource constraints in addressing complex diseases like cancer. Its application is vital for achieving Universal Health Coverage (UHC) goals, as it ensures that effective health interventions reach all segments of the population, particularly in developing settings where health disparities are most acute [14].

Implementation science frameworks are critical tools in health services research and delivery. They guide the systematic translation of research findings into practice, evaluation of health programs, and informing policy development and strategic planning [15].

This research aims to develop a stakeholder-informed guidance that could support effective implementation of the Global Platform for Access to Childhood Cancer Medicines and other childhood medicines access programs in Nigeria in general. This could help maximize the opportunities for such initiatives to deliver on their intended goals in LMIC settings.

## 2 Methods

### 2.1 Research framework

For this research, the “Consolidated Framework for Implementation Research (CFIR)” was used as it is particularly useful for assessing and guiding the implementation of health interventions, programs, and policies [16]. The CFIR framework provides a systematic approach to identifying factors that might influence implementation effectiveness, and consists of five major domains: intervention characteristics, outer setting, inner setting, characteristics of individuals, and process of implementation. The intervention characteristics address specific attributes of the intervention itself, such as its complexity, adaptability, and the quality of its design. The outer setting is the external context that could affect implementation such as patient needs and resources, policies, and incentives. The inner setting is the internal context of the setting or system where implementation of an intervention will take place such as readiness for implementation. The characteristics of individuals consider the individuals involved in the implementation and their beliefs, knowledge, self-efficacy, and attitude towards the implementation. The process of implementation covers the actual process of executing an intervention such as planning, engagements, and evaluation [16].

In the context of improving access to childhood cancer medicines in Nigeria through the Global Platform launched by WHO and St. Jude’s, the CFIR framework could be particularly useful in identifying and addressing factors at multiple levels, including the healthcare system and policy environment, readiness for implementation, the attitudes and skills of healthcare workers, and the strategies and steps needed for implementing the platform. This could help provide a comprehensive understanding of the factors that may influence the successful implementation of the platform in Nigeria, enabling targeted strategies to address potential barriers and leverage facilitators.

### 2.1.1 Study sample

Participants were recruited using the principles of the stakeholder categories framework [17] and reflected perspectives that were relevant to decision-making on access to medicines in Nigeria [18]. An initial sampling frame was developed and consisted of policymakers from the Ministry of Health, organizations implementing access to medicines programs in Nigeria, international organizations supporting the Federal Ministry of Health, medicines logistics providers, health professionals and personnel at service delivery points such as oncologists and pharmacists. Specifically, stakeholders were drawn from organizations including the Federal Ministry of Health, National Product Supply Chain Management Programme (NPSCMP/NSCIP), Clinton Health Access Initiative, BIO Ventures for Global Health (BVGH), International Association of Public Health Logisticians Nigeria Chapter, and federal tertiary hospitals that provide pediatric oncology services. Participants were senior officials in these organizations, and thus had the potential to provide relevant and rich data, and a wide range of valuable opinions and experiences.

The focus on these stakeholders was generally at the national and subnational levels, especially in relation to the inner setting of the CFIR framework. This general health system focus was due to two reasons: the inner settings of different institutions sum up the general health system inner setting; the internal plans of St. Jude’s and WHO are not in the public domain, thus, their preferences on implementing the platform and what institutions to involve are not clear.

### 2.1.2 Data collection

Data collection involved key informant interviews using a pilot-tested semi-structured interview guide. The pilot-testing was done with two staff working in the DRF unit of a North Central Nigerian hospital and a senior registrar working in an oncology unit in a South-West Nigerian hospital. Those that participated in the pilot-testing were not included in the main study. The development of the interview guide was informed by the CFIR framework and a comprehensive search of both peer-reviewed and grey literature from the Federal Ministry of Health, including white papers, policy documents, and press releases. The search focused on concepts related to access to pediatric oncology medicines in Nigeria and other medicines access programs in Nigeria such as the DRF. The resulting discussion guide explored the following key themes: lessons learnt from previous access to medicines programs; stakeholders and partnerships for success; and the four domains of the CFIR framework. Key informant interviews were conducted both virtually and in-person to reduce barriers to participation. The interviews took place from 21 June 2023 to 21 October 2023. The interviews lasted for 55 minutes on average. All interviews were conducted by the corresponding author. Based on participant’s oral informed consent, the interviews were recorded for transcription and coding. Throughout the data collection process, data saturation was assessed to ensure that theoretical saturation was achieved.

### 2.1.3 Data analysis

Data analysis was done by thematic content analysis to identify recurring themes and patterns in the data, in line with the interview guide. Recordings were transcribed verbatim, and the transcript subjected to open and axial coding using master sheets. Two individuals independently carried out the analysis to capture all possible different/variable perspectives and consensus meetings held to harmonize the data. An audit trail of the analysis process was kept, and all files related to the project are stored on a secure University of Toronto server. Results are presented through narrative synthesis, classified according to the themes that emerged, and supplemented with verbatim quotes. The authors also ensured reflexivity on how their background, experiences, and biases might shape the study, ensuring that these did not influence their interactions with participants and interpretation of the data.

Ethical approval was obtained from the National Health Research Ethics Committee of Nigeria and the Ethics Review Board of University of Toronto. Participants were adequately briefed about the research and its objectives. Participants were informed that their anonymity, confidentiality, and privacy will be maintained. They were also informed that data from the interviews will be used to inform the development of guidance for an implementation framework for the Global Platform for Access to Childhood Cancer Medicines for Nigeria and will be published. In line with the ethics process for this research, participants then gave oral informed consent prior to participation. Informed consent was documented in the researcher field notes and was not audio recorded as consent had to be obtained before recording can commence. At the end of the recording, participants were thanked for participating in the study.

### 2.1.4 Inclusivity in global research

Additional information regarding the ethical, cultural, and scientific considerations specific to inclusivity in global research is included in the Supporting Information (S1 Checklist).

## 3 Results

Eighteen (18) respondents participated in the study. Two of the respondents were Ministry of Health of officials familiar with the Cancer Access Partnership (CAP) and Cancer Health Fund (CHF) programs; one respondent was from NPSCMP/NSCIP; four respondents were from International Organisations supporting the Federal Ministry of Health such as Clinton Health Access Initiative and BIO Ventures for Global Health; three respondents were from the International Association of Public Health Logisticians Nigeria Chapter; five respondents were pediatric oncologists working in tertiary hospitals that provide oncology services; and three respondents were DRF staff in three different hospitals in Nigeria whom were all pharmacists.

The extensive conversations with various stakeholders provided insights into the current landscape and the future potential of improving access to childhood cancer medicines in Nigeria. Drawing from these discussions, key themes emerged for improving access to childhood cancer medicines in Nigeria. The results are presented according to the concepts of the CFIR framework. The results are informed by the participants perspectives on the current barriers in Nigeria with access to childhood cancer medicines, as well as challenges with existing access to medicines programs. Their perspectives were distilled to better understand what needs to be done differently to overcome the current barriers and avoid the pitfalls of the existing programs.

### 3.1 Outer setting

This refers to the external context affecting the implementation, including policy and regulations from governments, recommendations and guidelines, and patient needs and resources and the facilitators and barriers to meeting those needs [16].

#### 3.1.1 Policy support and financing for improving pediatric cancer treatment

Respondents noted weak policies for addressing pediatric cancer in Nigeria and the need for government commitment to enact new policies or review existing ones.

“*Pediatric cancers are not yet included in the National Health Insurance Scheme, which covers only a few adult cancers. This exclusion further limits access to necessary treatments for pediatric cancer patients.”*

This non-inclusion further exacerbates the financial burden of cancer treatment on families, driving inequities in access to care.

“*More inclusive financing models should be considered, such as expanding the scope of the National Health Insurance Scheme and establishing funds specifically for indigent patients… Alternative financing models should also be explored, including philanthropic models, public-private partnerships, and international funding platforms.”*

The need for exploring international funding platforms and public-private partnerships to support existing programs and infrastructure was highlighted.

“*We have the CAP model and Cancer Health Fund which can be integrated and then we include pediatric cancers into it. We can then involve local private sector players for non-clinical logistics and supply chain management support, enabling hospitals to access drugs without the burden of direct procurement. This way, we maximize funds and improve access to better-priced quality medicines.”*

#### 3.1.2 Weak policy affecting affordability and efficient resource use

The non-inclusion of childhood cancer medicines in the essential medicines list was noted to have led to off-label use of adult and childhood cancer medicines which could potentially harm patients and leads to waste, further straining a severely resource-constrained setting.

“*Some cancer medicines are used in both pediatric and adult oncology. But the issue we are having is with the dosage formulations. Medicines that are common to both may be listed on the essential medicines list, but typically in adult dosages. For instance, if pediatric oncology requires a 10 mg dosage, what we have available is a 100 mg adult dosage. This creates a problem for treating pediatric patients.”*

While pharmaceutical compounding was noted to be a solution, it still has its challenges of availability, quality, and resource wastage as some facilities lack compounding units and extra quantities of preparations from adult dosages could go to waste. Thus, hospitals could consider ways of central dispensing and pooled compounding.

#### 3.1.3 Streamline legal and regulatory frameworks to address bureaucratic bottlenecks

Strengthening the legal and regulatory framework and addressing bureaucratic bottlenecks to support initiatives for improving access to childhood cancer medicines, including import approvals, pricing controls, and addressing complex regulatory bureaucracies were highlighted as necessary. A significant challenge noted is the reluctance of large pharmaceutical companies to distribute and market childhood cancer medications in Nigeria. This reluctance is attributed to regulatory hurdles with the National Agency for Food and Drug Administration and Control (NAFDAC) and purchasing power of patients.

“*Large manufacturers of oncology medications do not have pediatric oncology medications in Nigeria because it is difficult getting the medicines in. Sometimes you spend much more than the cost of the medicine in the process of getting them into Nigeria. How much, realistically would you be able to sell it? How many people would be able to afford these medicines?”*

This regulatory difficulty in importing products was also highlighted by civil society organizations working in access to medicine in Nigeria.

“*One example is when we had a donation of light microscopes from [name removed] because they were shutting down a facility in the United States. Our partners in Nigeria were very keen to obtain these valuable and almost new microscopes to advance some of their pathology work and training. It took us more than two years to get those microscopes imported into Nigeria with a lot of back and forth on waivers. They told us we needed an acknowledgement from the Ministry of Health to be sent to the Ministry of Finance. And then there is someone in the Ministry of Finance that needs to sign off a form and then that needs to be back to the Ministry of Health, and they need to send an acknowledgement to the Nigerian Customs Service, which also needs another acknowledgement, and so on.”*

Such bureaucracy and inefficiencies were noted as frustrating and deters individuals and organizations that are willing to help, leading them to “just give up”.

#### 3.1.4 Patient-level challenges and community support

Respondents highlighted that socio-economic factors, cultural perceptions, and geographical barriers significantly impact access to care and medicines for childhood cancers. Lack of financial empowerment significantly hinders access to expensive cancer treatments, as most people cannot afford these medications.

“*In my long career, I have only seen insurance cover two patients for oncology treatments and this coverage was by private insurance. Almost all the parents of the patients we see cannot afford to pay for the drugs needed to treat their children. Many times, we contribute money to help some of these patients but there is only much we can do. The fact that health insurance coverage is almost non-existent does not help these patients who continue to bear the brunt of high medical expenses.”*

Respondents also noted geographical disparity and variation in access across the country, with urban centers having better access than rural areas. In many cases, practitioners in rural areas often rely on their counterparts in urban centers to source medications.

Patients in rural areas also face significant travel burden, with patients often traveling long distances for care, especially when certain medicines and specialized treatments like radiotherapy are not available locally. In addition to the high cost of the treatments, they also bear costs for transportation and accommodation, and risks associated with travel in Nigeria.

“*Many of our patients have to travel to Lagos or Abuja to get certain treatments; and because they are children, they have to travel with their parents or guardians. This places huge burden on both the patients and their families, in terms of time, cost, and logistics.”*

These challenges make even the most modestly priced medicines to become out-of-reach. Thus, since programs like the platform for improving access to childhood cancer medicines may not be able to directly enhance the economic power of individuals, their design should consider ways to ensure that their implementation does not further widen access gaps. The need for community awareness and education to dispel myths about cancer and promote early detection was also highlighted.

“*Many of the patients we see present late because of many reasons such as myths and misconceptions about cancer. There is general fear about cancer which is often seen as a death sentence. But these communities do not know that childhood cancers have better survival chances. The platform needs to include components for increasing community awareness about childhood cancers and educating the public to help dispel myths and misconceptions, promote early detection, and reduce treatment abandonment.”*

The need for support systems for patients and their families was highlighted, including provision of financial aid and palliative care services, and creating networks for families dealing with childhood cancer for mutual support and resource sharing. It was suggested that community awareness programs should engage community leaders, religious and traditional leaders, civil society, and the use of local media channels. These stakeholders can also help in community advocacy and mobilization, ensuring community buy-in and support for the program. Their advocacy can also be extended to political leaders in terms of putting pressure for the right policies and regulations to support the program and improve sustainability.

### 3.2 Inner setting

This domain examines the internal context within the organization where the intervention is being implemented. It includes aspects like the organization’s culture, implementation climate, and readiness for implementation [16].

#### 3.2.1 Addressing corruption and enhancing governance

Respondents highlighted corruption as impacting overall efficiency and the integrity of health service delivery. Corruption often manifests in administrative processes and leads to misappropriation of funds and wider systems inefficiencies which affect the availability of medicines. It causes lengthy delays in paying suppliers and vendors which causes supply chain disruptions thus leading to stockouts of essential medical supplies.

Addressing corruption will ensure that leakages are blocked, and funds are channeled for the appropriate use, including procurement and payment of suppliers. This will ensure that expectations are met across all players involved in ensuring that medicines are available and accessible.

“*Just the way we expect suppliers to deliver quality products on time, suppliers similarly expect timely payments. This reciprocal relationship is crucial to maintain a healthy procurement and supply chain system for these medicines so that patients can benefit. It will also help in better pricing because if the supplier knows that you will pay on time, they do not need to sell at high mark up which they do to account for delays and inflation. It will also help them in balancing demand and supply which also helps with pricing.”*

Addressing corruption also requires setting up effective governance mechanisms and strengthening existing ones such as the Drug Management Agencies (DMA). These mechanisms can support independent decision making and operations within a defined scope, in addition to technical capacity to ensure accountability. DMAs can serve as centralized and accountable entities, leading to less fragmentation, and better streamlining of processes.

#### 3.2.2 Existence of programs to improve access to cancer medicines

The existence of programs for improving access to cancer care was highlighted, with respondents suggesting that the platform can leverage on them to improve access to childhood cancer medicines. There are two government-partnered cancer care programs in Nigeria: CAP, which offers subsidized oncology drugs through a partnership between the government and pharmaceutical companies, and the Cancer Health Fund (CHF), a government-funded program that covers investigation and treatment costs for specific adult cancers (prostate, cervical, and breast cancer).

However, challenges with these programs were noted. For instance, CHF requires patients to meet certain criteria to be considered indigent and eligible for free treatment, which may also limit access to other people who need these treatments but cannot pay. CAP, while offering significant discounts on oncology medicines, does not cover all types of cancer medications, limiting its effectiveness in providing comprehensive access. Even with substantial discounts (around 35–40%), the high cost of oncology medicines means that patients still face considerable out-of-pocket expenses, which many find challenging to afford. The program’s reach is currently limited to certain hospitals, with a focus on expanding to facilities that have a multidisciplinary team for cancer care. Another major issue highlighted is the frequent unavailability of drugs, despite the government’s partnership with pharmaceutical companies. Finally, there is a lack of specific provisions or additional support for childhood cancer patients.

However, it was noted that despite the challenges of these programs, including lack of focus on childhood cancers, their structure can be leveraged and enhanced to improve access to medicines for childhood cancers.

“*We are currently advocating for expansion and inclusion of pediatric cancers and to cover more hospitals. This means the platform could not have come at a better time and can help us achieve this inclusion which can also help improve the sustainability of the program.”*

#### 3.2.3 Data management and monitoring

The existence of data management and monitoring systems, even though they are fraught with challenges was also highlighted. While some respondents noted that effective data management systems are crucial for accurate forecasting, tracking medicines availability, and evaluating program impact, they noted that there are limitations as data is processed mainly through registries with limited entry points, making it difficult to use for forecasting medicines needs or understanding patient outcomes. National reporting systems like DHIS2 were noted as often tracking only a few types of cancer medicines, leading to a significant gap in planning for other cancers, such as childhood cancers.

However, there are newer systems in place which help with managing logistics data for medicines, such as the Nigeria Health Logistics Management Information System (NHLMIS). CAP also has a digital platform used for distributing and tracking cancer medicines. Respondents suggested leveraging these systems to streamline the logistics and supply chain process for childhood cancer medicines instead of creating new ones.

### 3.3 Characteristics of individuals

This domain considers the individuals involved in the implementation and their beliefs, self-efficacy, knowledge, and attitudes towards the intervention [16].

#### 3.3.1 Human resource constraints and the need for capacity building

Respondents noted that there is a critical shortage of specialist or specialized human resources across all levels of the healthcare system, from the national/tertiary level to the local government/primary care level. This shortage is exacerbated by the flight of human resources to countries with better economic situations and conditions for healthcare workers than Nigeria. At the primary care level, specialists are lacking, and human resources are much more depleted. This leads to a gap in knowledge of childhood cancers, with patients making multiple visits before cancer is suspected, leading to delays in diagnosis and treatment. Even in secondary and tertiary facilities, the appropriate human resources are lacking. General practitioners and medical officers often take up roles in oncology clinics even without appropriate training in oncology. Clinicians were identified as often doubling up as public health planners, implementers, and evaluators.

These challenges affect the early detection and treatment of childhood cancers, with some respondents suggesting that addressing the shortage of specialized healthcare professionals and enhancing training for existing staff is crucial. This includes developing local training programs and improving the capacity of primary healthcare workers for early recognition and referral of childhood cancer cases for proper management and treatment. Some respondents also suggested task-shifting and training of lower cadre health workers to manage simpler cases as a viable strategy to improve the cancer workforce. Furthermore, some of the pediatric oncologists called for training and institutionalization of oncology pharmacists as they are key players in managing the access, distribution, and administration of childhood cancer medications.

“*Many of the pharmacists we have do not have specialist training in oncology. We need them to be trained to help us provide expert opinion and professional counseling on rational medicines use for childhood cancers. They can also support in other areas beyond medicines access including health promotion and education within communities, and can also support in some aspects of screening, diagnosis, and treatment.”*

Some respondents highlighted the need for in-country training programs for everyone involved in the care and treatment of childhood cancers. Most of the training in childhood cancers are offered abroad, amidst prohibitive costs that make it inaccessible for potential trainees.

“*Many of us that work in pediatric oncology in Nigeria do so out of personal passion or interest, rather than formal training or adequate incentives. We collaborate with adult oncology teams for training and skill development; but this should not be a substitute for formal, specialized training in pediatric oncology.”*

#### 3.3.2 Improving primary care

Respondents also highlighted the need to focus on primary healthcare as a foundation for improving access, recognizing symptoms of childhood cancer, and making timely referrals. Training community health workers and primary care physicians in basic oncology care can facilitate early detection and initial management. Incorporating childhood cancer awareness into primary health care activities was also suggested.

#### 3.3.3 Government involvement

Respondents highlighted the central role of government in driving and coordinating efforts for improved access to childhood cancer medicines. Some improvement in the attitude of government towards the recognition of childhood cancers was noted, which was achieved through strong advocacy efforts and policy influence for increased funding and prioritizing childhood cancer in the national health agenda.

“*Pediatric oncology has not been a priority for the Nigerian government. Our efforts to include pediatric oncology in national health plans were initially overlooked. But we are seeing some changes manifest, and the government is beginning to engage more with us. The Nigerian Society of Pediatric Oncology is now being invited to important meetings by the government. We [pediatric oncologists] were recently invited to participate in the review of the cancer control plan and the inclusion of dedicated pediatric oncology sections in the plan.”*

Some international partners such as the Clinton Health Access Initiative and BVGH were identified as being instrumental in advocating for the inclusion of childhood cancers in the national agenda in Nigeria. Additionally, the recent launch of the National Institute for Cancer Research and Treatment was noted as a significant step towards consolidating efforts in cancer care. Thus, there is some optimism about the recent developments and the growing attention to childhood cancers, both nationally and internationally, which positions Nigeria to benefit from international initiatives for improving childhood cancer care.

It was noted that these efforts are crucial for changing the global perception of Nigeria’s capacity and involvement in childhood cancer access programs such as the Global Initiative for Childhood Cancer and the platform by WHO and St. Jude’s. Nigeria has significant patient volumes, thus support and resources from international organizations are highly needed.

### 3.4 Intervention characteristics

This domain focuses on the specific attributes that should be considered for the intervention, such as stakeholders’ perception of the approach to implementation, the degree of adaptation to meet local needs, and the method and quality of the intervention’s design [16].

#### 3.4.1 Partner-driven approach and integration into existing systems

A partner-driven approach for implementing the platform in Nigeria was suggested, including partnerships with international organizations working to improve access to medicines, government, hospitals, and communities, and leveraging their already existing expertise, resources, and infrastructure. Engaging partners in Nigeria to understand the local needs and priorities and avoiding imposition of foreign development ideas and solutions that may not be relevant to the Nigerian context was buttressed. This could reduce the tendency for duplication of efforts associated with running parallel programs with similar aims, strengthen existing systems such as CAP and CHF, thus optimizing resources and improving efficiency. It was also suggested that healthcare professionals, communities, and patients should be engaged to ensure that the interventions are culturally acceptable.

“*Any organization implementing the platform should approach the Nigerian healthcare system with humility and respect, acknowledging the local healthcare professionals’ expertise and challenges in delivering care to pediatric cancer patients. The platform should support and work alongside local professionals, many of whom have built a rapport with the communities they work in and understand cultural contexts, rather than imposing external methods or bringing in new partners unfamiliar with Nigeria’s healthcare landscape. They should not go in with the attitude that, we are the ones that know how to treat pediatric cancer patients and you don’t… [This] could cause disruption and affect the sustainability of the platform.”*

They also suggested that the platform should also look at community and patient-level engagement and education to ensure that patients do not abandon treatment. Community-based organizations should also be partnered with to support information dissemination, demand generation, patient navigation and treatment support.

#### 3.4.2 Delivery strategy for the platform

Respondents gave suggestions on how best to deliver the platform in Nigeria based on experiences from similar efforts aimed at improving access to medicines in the country. The recurring strategy was to adopt a pilot and scale approach by selecting a few hospitals to pilot the platform, drawing from hospitals that have performed well in implementing DRF. The platform can then be gradually scaled based on the insights gained from the pilot. Due to lack of clarity on the exact approach the platform intends to use, some respondents suggested ensuring robust data management systems, improved procurement practices to ensure cost efficiency, and consideration of economic realities to ensure affordability. Improved procurement practices and strategic purchasing could improve efficient price negotiation. This approach was highlighted as having the possibility to stimulate supplier interest by creating a commercially viable market beyond just social responsibility or outreach. Some respondents also highlighted the need for strategic sourcing and supplier management which allows for better anticipation of needs and more effective planning throughout the supply chain, ultimately impacting the cost and availability of products.

“*Some of these considerations are crucial for offering these medicines at the right price. Please note that I said the right price and not necessarily the lowest price because the right price balances affordability for patients with the need to sustain healthcare services and supply chains. It is not just about reducing prices or free medicines but considering the broader economic context and the purchasing power of individuals, in addition to the need for ensuring sustainability and that the program continues running.”*

Further on sustainability, concerns were raised on the lack of robust information about country selection and platform implementation, whether the platform is a one-time initiative or an ongoing funding stream, the later, which will be crucial for long-term planning.

Some respondents also highlighted the need to balance planning and action as nothing much has been heard about the implementation of the platform since its announcement in 2021. They highlighted the need to move beyond the planning stage to taking concrete actions, as patients, especially children, urgently need access to these medicines.

“*Get started. You are going to learn as you go. While you want to have a good framework and system and very strong partners, get the platform started. Children need access to these medicines and Nigeria needs impact and success in cancer patient management. So, you need to stop planning at some point, and you need to start doing. And I think all the patients and their families would agree.”*

### 3.5 Process of implementation

This covers the actual process of implementing the intervention, including planning, engaging, executing, and reflecting and evaluating [16].

#### 3.5.1 Integration into existing programs, including maternal and child health programs to leverage on existing structures and established trust and familiarity with communities

Integrating the platform into existing programs and structures was a recurring theme amongst the respondents. Implementing the platform within the framework of existing structures such as DRF, CAP, and CHF could ensure that there is less duplication and more coordination, enhancing uninterrupted access to childhood cancer medicines and sustainability of the platform. Some suggested that the platform could link its implementation to maternal and child health programs since the focus is on childhood cancers, and that this approach could also help improve awareness and screening for early detection of childhood cancers. This approach could capitalize on established trust and familiarity within communities to ensure smooth implementation and adoption of the platform.

#### 3.5.2 Using a project management approach that involves stakeholder collaboration and change management

Respondents also suggested ensuring efficient project management that involves setting clear goals, timelines, and milestones for the platform. This should involve collaborating with stakeholders on the ground to develop a long-term vision for not just the platform, but also for childhood cancer care in Nigeria. This was suggested as being crucial to ensuring sustainability and guiding the scalability of the platform to different contexts within Nigeria and tailoring various components of the intervention to specific needs and capacities of different states and communities. Furthermore, this strategy of setting clear goals and collaborating with stakeholders was deemed necessary to keep stakeholders motivated and accountable for their contributions, which could lead to better outcomes for the platform. Some respondents also suggested that this could help in engendering change management aimed at addressing weaknesses within the system by preparing, supporting, and helping individuals in the system to successfully adapt to changes thus limiting resistance to change.

#### 3.5.3 Feedback mechanisms and continuous improvement

Respondents also suggested that establishing mechanisms for regular feedback from patients, families, and healthcare providers can identify gaps and areas for improvement. Continuous monitoring and evaluation of the platform should be carried out to adapt and refine strategies based on real-world outcomes and feedback.

#### 3.5.4 Stakeholder collaboration and coordination

Respondents suggested the need for collaboration among various stakeholders, including patients, government and its agencies, health workers, civil society, international partners, and even pharmaceutical companies.

Patients and communities were identified as primary stakeholders and efforts should be geared towards creating awareness and building trust, in addition to educating them to dispel myths, address stigma, and improve understanding on the significance of early diagnosis on better treatment outcomes. Involving community leaders and faith-based organizations was regarded as key as their influence and proximity to the community makes them vital in disseminating correct health information and encouraging acceptance of health interventions.

The government is also a primary stakeholder and the central coordinating body necessary for political support, and addressing weaknesses in policy, regulation, infrastructure, and resources. Agencies like NAFDAC and the Nigerian Customs Service are crucial for licensing and approving oncology medications entering Nigeria, ensuring they meet the regulatory standards, and that the process for obtaining necessary import waivers and clearance procedures is seamless. The National Health Insurance Authority is crucial for ensuring insurance coverage of childhood cancer medicines and treatments. The National Cancer Control Program is crucial for coordinating cancer-related initiatives and can assist in liaising with other government entities and pushing for the inclusion of childhood cancer medicines into the essential medicines list.

Health workers are crucial for the daily implementation of the program, and opportunities and resources for additional training on childhood cancers should be provided to them. The Body of Chief Medical Directors and Medical Directors were also deemed crucial partners for implementing multidisciplinary teams within tertiary hospitals, which are often the primary centers for cancer treatment. Health workers’ professional bodies such as the Nigerian Medical Association, the Pharmaceutical Society of Nigeria, and the National Association of Nigeria Nurses and Midwives are crucial to providing support in terms of advocacy, guidance, and ensuring the engagement of health workers across different specialties.

Civil society organizations such as the Nigerian Cancer Society, Nigerian Society for Pediatric Oncology, and parents and patient advocacy groups such as the Dorcas Cancer Foundation and Okapi Children Cancer Foundation, are crucial for advocacy, building community trust and acceptance, and countering misinformation. International partners, such as the Clinton Health Access Initiative and BVGH have expertise in childhood cancer medicines access and could be invaluable for navigating potential challenges and addressing past mistakes and challenges in implementation. Pharmaceutical companies are also crucial for ensuring competitive drug pricing and constant availability of medicines.

The need for the platform to engage the private sector was highlighted, given the additional support they could lend to the platforms sustainability through corporate social responsibility.

Respondents generally highlighted the need for a multi-stakeholder approach, which is essential for navigating the complex landscape of healthcare delivery, regulatory compliance, and community engagement. Measures for success were highlighted including increased availability, affordability, accessibility, and acceptability, completion of treatment courses by patients, improved patient care and experience, and overall improvement in treatment outcomes.

Respondents believe that Nigeria has what it takes to implement the platform even though support is needed to address some of the challenges to ensure smooth implementation.

“*Overall, we have the basic structure and components required to implement the platform. What we need to do is to address some weaknesses, most of which can be done through proper governance and accountability measures, tightening loopholes, and then followed by scale up and institutionalization of best practices to ensure access to these medicines.”*

## 4 Discussion

This study aimed to inform the development of an implementation framework for improving access to childhood cancer medicines in Nigeria aligned within a structured implementation science framework–the CFIR framework–to interpret the data [16]. The findings reveal critical insights spanning five domains of the CFIR framework, each contributing uniquely to understanding the complexities of childhood cancer medicine access in Nigeria with a view to understanding pathways to implementation of the platform and improving access.

For the platform to be successfully applied to LMIC contexts like Nigeria and to address challenges in access to childhood cancer medicines, findings suggest the need for collaborative, partner-driven approaches, and integrating the platform into existing healthcare infrastructure and programs with similar aims. In Nigeria, there is CAP and CHF. There is also DRF which is in existence in many other LMICs in Africa. DRF is a cost-recovery mechanism that ensures the provision of high-quality medications directly from producers at reasonable prices by cutting off middlemen. Following an initial investment, the revenue generated from medicines sales is used to restock the supplies [18]. CAP, on the other hand, is a collaboration with various pharmaceutical firms to supply quality cancer medications at reduced costs to treatment centers in sub-Saharan Africa [19]. CHF, a collaboration between the government, Roche, and the International Finance Corporation, aims to improve affordability of quality cancer care and treatment for economically disadvantaged groups by covering costs of treatment [20]. This collaborative strategy emphasizes the importance of leveraging existing resources and expertise to avoid duplication of efforts and inefficiencies. Evidence suggests that in Nigeria, poor donor coordination and collaboration has led to duplication of efforts across healthcare projects and platforms, leading to waste and inefficiencies in resource use [21]. This issue of duplication of efforts and it cost implications in health service delivery have also been reported in high-income settings [22]. For interventions such as that provided by the platform, there is need for partnership synergy and consideration of mechanisms that promote meaningful partnerships including building trust between partners and having belief in the collaboration to boost performance [23].

The study also revealed critical policy and financing gaps that impact access, notably the exclusion of childhood cancers from national health plans and insurance schemes which exacerbates the financial burden on families. Findings also showed regulatory challenges and geographic disparities affecting access to care, in addition to health workforce shortages, systemic corruption and poor governance. These findings have been reported in the literature as beleaguering the implementation of projects in the healthcare sector in Nigeria and hampering health sector improvements [24–26]. While the onus lies on government, its agencies, and concerned in-country stakeholders to fix these problems, donor funded programs such as the Global Platform for Improving Access to Childhood Cancer Medicines could be crucial for driving reform. The platform could put additional pressure and lobby for health system changes using their negotiation advantage derived from the unique support they are bringing to the country. Evidence supports this, especially in cases where such settings have shown willingness and are already adopting changes [27]. This is the case in Nigeria where childhood cancers are being considered for inclusion into the national cancer control plan. Also, according to the findings, childhood cancer specialists are becoming increasingly included in conversations for addressing cancer burden and mortality in the country. Evidence also suggests that leadership and planning investments by donors in a country’s financial, technical, and logistical capacity are crucial for ensuring sustainability beyond the duration of a project [28] so long as it does not undermine national capabilities.

The CFIR framework is widely recognized for its broad and comprehensive nature as it covers a wide range and depth of data effectively [29]. However, this comprehensive scope acts as a double-edged sword as it may be too broad to ensure all constructs are fully explored within the constraints of typical interview durations [29]. Therefore, its effectiveness is enhanced through multiple interviews based on a focused approach [29].

This study has accounted for this by conducting multiple interviews amongst various stakeholders, focusing on issues to consider in implementing the platform. The framework proved instrumental in highlighting issues to be considered in implementing the platform in Nigeria, offering a structured lens for viewing various factors influencing the platform’s adoption. However, it is still necessary to suggest a nuanced implementation framework for the platform in Nigeria which might help in providing a more contextualized and localized adaptation of how the implementation of the platform should be approached. Therefore, based on our findings, we developed a stakeholder-informed guidance for the platform as shown in Table 1.

**Table 1:** Proposed guidance for implementation of the Global Platform for Improving Access to Childhood Cancer Medicines in Nigeria.

	Domain				
	Intervention Characteristics	Outer Setting	Inner Setting	Characteristics of Individuals	Process of Implementation
**Implementation**	Follow a partner-driven approach fostered by consultations with stakeholders on the ground and leveraging existing platforms to streamline implementation. *(key actors to consider*: *DRF*, *CHF*, *CAP program structures; other development partners working in cancer such as CHAI and BVGH; federal and state ministries of health)*	Consider support systems for patients and families, accounting for transportation, accommodation, and financial assistance, to address geographical disparities and financial constraints caused by out-of-pocket expenditure and poor insurance coverage. *(key actors to consider*: *civil society*, *patient groups and advocacy networks)*	Support efforts in promoting health system integrity and addressing weak governance and corruption by adopting newer mechanisms being put in place such as DMAs into the implementation of the platform. *(key actors to consider*: *federal and state ministries of health; heads of hospital administration; oncology workforce; DRF*, *CAP*, *CHF mechanisms; international development partners)*	Support comprehensive training for healthcare providers in childhood cancers, including the latest treatment protocols and patient management techniques. *(key actors to consider*: *federal and state ministries of health; oncology workforce)*	Adopt a project management approach by developing a clear, actionable plan involving key stakeholders such as government agencies, healthcare providers, NGOs, and patient advocacy groups. *(key actors to consider*: *DRF*, *CHF*, *CAP program structures; other development partners working in cancer such as CHAI and BVGH; federal and state ministries of health; civil society; patient advocacy groups; oncology and other health workforce)*
	Work with locally based logistics partners to ensure constant product availability and distribution. *(key actors to consider*: *medicines logistics providers; NHLMIS; International Association of Public Health Logisticians Nigeria Chapter; National Supply Chain Integration Program)*	Work with the government to develop policies that support, not only childhood cancer treatment and medication subsidization, but address regulatory and bureaucratic bottlenecks. *(key actors to consider*: *federal ministry of health and concerned agencies; policymakers including the legislature)*	Engaging with existing programs with similar targets such as CAP and CHF to use their knowledge and experiences in implementation and build synergy for addressing mutual concerns and interests. *(key actors to consider*: *DRF*, *CAP*, *CHF mechanisms; international development partners like CHAI and BVGH)*	Support alternative strategies to workforce development, such as task shifting, and support the inclusion and use of available health workers that could support childhood cancer care. *(key actors to consider*: *federal and state ministries of health; oncology workforce; other health workers)*	Integrate the platform into existing programs, leveraging existing structures and established trust and familiarity with communities. *(key actors to consider*: *DRF*, *CHF*, *CAP program structures; other development partners working in cancer such as CHAI and BVGH; federal and state ministries of health)*
	Consider socioeconomic factors in price determination and adopt discounted pricing strategies. Consider assessing existing in-country suppliers to determine possibility of their inclusion as part of strategic sourcing and management strategy which could enhance constant product availability and better pricing. *(key actors to consider*: *CAP and CHF mechanisms; international development partners like CHAI and BCGH; pharmaceutical companies and suppliers)*	Leverage local and international partnerships for advocacy and knowledge transfer to address myths around childhood cancers and improve community support. *(key actors to consider*: *civil society; community support groups; international development partners; patient families and care givers; communities)*	Support strengthening, integration, and use of in-country data management and monitoring strategies such as NHLMIS and CAP’s digital platform to track and monitor medicines and make data-driven decisions. *(key actors to consider*: *federal and state ministries of health; NHLMIS; National Supply Chain Integration Program; DRF*, *CAP*, *CHF mechanisms)*	Support capacity strengthening at the primary care level to improve childhood cancer symptom recognition and early referral. *(key actors to consider*: *federal and state ministries of health; oncology workforce; other health workers)*	Implement the program with ongoing monitoring and evaluation mechanisms to assess effectiveness, identify areas for improvement, and adapt strategies as needed.
	Utilize globally recognized treatment guidelines adapted to the local context and providing support to relevant policy stakeholders in developing a contextualized treatment protocol and inclusion of childhood cancer medicines into the essential medicines list. *(key actors to consider*: *federal and state ministries of health*, *their relevant agencies such as National Institute for Cancer Research and Treatment (NICRAT)*, *and their formulary committees)*		Support relevant stakeholders to leverage the recent attention of government on childhood cancers to drive further leadership engagement for innovation and change in the neglected aspects of childhood cancer care. *(key actors to consider*: *Relevant government agencies like NICRAT; civil society; patient advocacy groups)*	Support patient and community awareness and education about childhood cancer, its signs, and available treatments; collaborating with relevant stakeholders like civil society and traditional/religious leaders. *(key actors to consider*: *civil society; community support groups; international development partners; patient families and care givers; communities)*	Establish regular feedback channels among all stakeholders, including patients and healthcare providers, and use this feedback for continuous improvement of the program. *(key actors to consider*: *DRF*, *CHF*, *CAP program structures; other development partners working in cancer such as CHAI and BVGH; federal and state ministries of health; patients and patient groups; oncology and other health workforce)*
	Adopt an incremental approach starting with pilots in few facilities to assess feasibility and effectiveness before scaling up. *(key actors to consider*: *facilities with better performance in implementing DRF; other development partners with existing structures such as CHAI and BVGH)*				Recognize, and not disregard, in-country capacity, focusing on ways to use and improve already available capacity, infrastructure, and systems.
	Balance planning with action and prioritize settings with highest burden of childhood cancers. Using burden of childhood cancers as major determinant of regions to implement the platform, while providing technical support to improve areas of perceived weaknesses.				Explore opportunities to encourage local manufacturing of cancer medications to reduce dependency on imports and lower costs. *(key actors to consider*: *federal government; pharmaceutical manufacturers)*

This table was developed according to the CFIR Implementation Science framework.

### 4.1 Limitations

While this study has strengths in terms of drawing insights from a wide array of stakeholders relevant to improving access to childhood cancer medicines in Nigeria, there were some limitations. The limited background information on the mechanisms of the platform beyond the general information available online, made it difficult to contextualize the findings within planned mechanisms of implementation by the organizers of the platform. Thus, participants were commenting based on their experiences with pitfalls of existing programs that ideally should be avoided when implementing the platform in countries like Nigeria. Furthermore, we could not obtain additional information from the organizers which could have helped in understanding their implementation plans and the factors to be considered in choosing countries and institutions for implementation. This was also a major criticism of the platform by respondents as since its launch, major movements for implementation seem not to have been made. Nigeria has one of the highest burdens of paediatric cancers and yet there has not been much known about when the platform will be implemented in Nigeria. Some respondents believe that the reason behind the delay and the lack of publicly available information on the platform is the skepticism about country-specific implementation contexts. Therefore, we hope that the findings of this study can help provide relevant information and insights that could be useful for implementing the platform in countries with challenging healthcare landscapes such as Nigeria.

## 5 Conclusion

This study developed a stakeholder-informed guidance to support effective implementation of the Global Platform for Access to Childhood Cancer Medicines in countries like Nigeria and maximize the opportunities for the initiative to deliver on its intended goals. Also, the challenges in pediatric oncology in Nigeria were identified which have implications for medicines access for childhood cancers. Understanding these challenges that need to be addressed may be worth considering for an implementation framework to be effective in addressing access to medicines and care for the disease.

The results provide insights from stakeholders on the areas of focus for the platform’s implementation to be successful. Results also highlighted the existence of systems that the platform can leverage on to pilot and scale the implementation. Using the proposed guidance could be beneficial for countries like Nigeria that continue to bear a disproportionately high burden of childhood cancers and ensure access for children who desperately need childhood cancer medicines to survive.

## Supporting information

S1 Checklist Inclusivity in global research questionnaire. (DOCX)
